# Editorial: Rhizosphere Manipulation for Sustainable Agriculture

**DOI:** 10.3389/fpls.2024.1378088

**Published:** 2024-02-16

**Authors:** Ishwar Prakash Sharma, Krishna Giri, Narendra Kumar

**Affiliations:** ^1^ Herbal Research Division, Patanjali Research Foundation, Haridwar, Uttarakhand, India; ^2^ Indian Council of Forestry Research and Education (ICFRE), Dehradun, Uttarakhand, India; ^3^ Forest Research Institute (FRI), Dehradun, Uttarakhand, India

**Keywords:** agriculture, rhizosphere, endophytes, technology, yield

The agriculture industry is now facing the challenge of addressing the issues of boosting productivity to feed the world’s expanding population and improving resource use efficiency while lessening the adverse effects of agriculture on ecosystems and public health. Chemical pesticides and fertilizers guarantee year-round productivity and maximize yield in both favorable and unfavorable situations, but they are expensive, reduce soil fertility after misuse, and exacerbate environmental pollution, all of which jeopardize the security of the world’s food supply. Over the last three decades, several technological advancements have been made to improve agricultural production by reducing the use of synthetic agrochemicals. Studies on the rhizosphere have recently made significant strides towards sustainable agriculture. The examined soil microbiota has been pivotal in plant growth and development. However, the rhizosphere’s composition, structure, and functions still require further state-of-the-art studies to exploit its potential to boost agricultural productivity. Despite historical methods of adding organic matter and inoculating plant growth promoting rhizobacteria in agriculture, there is very limited scope for rhizosphere manipulation. Tillage operations and other inputs such as herbicides, fertilizers, and irrigation are typically used to modify the rhizosphere in agricultural land. Recent scientific advances—such as novel gene editing, multi-omics methods, and synthetic biology techniques—and many datasets, including the transcriptome, metabolome, proteome, and genome, have been used to modify the rhizosphere.

Modifications in the rhizosphere and rhizospheric biota have tremendous potential to alleviate the adverse impacts of high input-based agricultural practices and achieve agricultural and environmental sustainability. A robust rhizosphere contributes to the health and productivity of the plant, which may then be altered and exploited to increase productivity. Plants are proficient in sculpting the microbiome that they are connected with. In the last several years, attempts have been made to alter the rhizosphere according to the requirements. Rhizospheric engineering aims to comprehend to improve agricultural yield, sustainability, forecast, and regulate rhizospheric environment and interactions. In light of the rising demands for organic food and farming and environmental sustainability, novel strategies must be developed. Therefore, rhizosphere modification is a cutting-edge method in modern agriculture to increase the output. The Research Topic on rhizosphere manipulations for sustainable agriculture aimed at compiling the most recent scientific advancements in crop production, recent findings, patterns, and inventions, various methods and approaches in rhizosphere engineering, and manipulation for increased agricultural productivity and sustainability.

The rhizospheric microbiota is beneficial for agricultural sustainability individually and as a community. The non-rhizobial endophytes are the active colonists in the root nodules whose roles are yet to be unraveled. For the first time, Debnath et al. described the role of non-rhizobial endophytes from lentil root nodules have been isolated and evaluated for their plant growth-promoting traits, such as the presence of *nifH* and *nifK* elements, exopolysaccharide, biofilm formation, and root metabolites. The emergence of root hair is attributed to their strong ability to colonize within the roots. While comparing the uninoculated and inoculated plants, these non-rhizobial microbes have markedly increased the exudation of triterpenes, fatty acids, and their methyl esters, changing the composition of the rhizospheric microbial population. These non-rhizobial endophytes can improve soil nutrient status, modulate rhizospheric microbiota, and increase the relative abundance of other rhizospheric microorganisms, leading to increased agricultural productivity.

Additional noteworthy findings include that many plants, mostly noxious weeds, have a robust rhizospheric microbiome to withstand harsh environmental conditions. In the rhizosphere of *Lantana camara* L., Gola et al. carried out Illumina-based metagenome sequencing and examined the rhizosphere diversity and the rhizospheric soil structure, which indicated a higher presence of rhizospheric microorganisms. These microorganisms offer plants superior stress response and growth promotion because they are well-adapted to stress conditions. The active compounds in the plant are produced more effectively when the root system is manipulated using these strategies. The active components of the plant are crucial to human civilization and are isolated from several commercial crops used in dye, sauces, medicines, and other products. Due to decreased soil fertility, changing climate, increasing urbanization, increased food consumption, etc., important commercial plants are presently in danger and must be preserved. Manipulating the rhizosphere is a sophisticated technique for maintaining and improving the yield of commercially relevant plants. Since aquaponics often uses aqueous conditions, it is essential to make careful adjustments to the solid waste since too much of it raises the BOD, which lowers the oxygen levels in the rhizosphere and causes ammonia and nitrate to build up, which are harmful to plant development. Singh et al. estimated the production of active components i.e., withaferin A and withanolide A in *Withania somnifera* (L.) Dunal used the aquaponics method and observed significant enhancement of these compounds. This method may be crucial for establishing integrated agricultural and food production systems, particularly in dry or desert regions where water is scarce. To enhance the development and production of *Curcuma longa* L., Khan et al. reviewed and explained the rhizospheric interactions in detail. The enormous synthesis of phytoconstituents like curcumin and improved plant development is dependent on the microbiota linked to the roots of *C. longa*.

By controlling the synthesis of primary and secondary metabolites, microorganisms, mainly those that promote plant growth, are essential to the growth and development of plants. Due to environmental issues brought on by the misuse of the broadcasting method, the rhizosphere, in addition to the microbiota, regulates the amount of nutrients in the soil, which is crucial for crop development. Swain et al. study the rhizospheric based phosphorus (P) fertilizer management and compare it with the broadcasting method in Chilli (*Capsicum annuum* L.) under alkaline soil. When the seedling roots are treated with the seedling root-dipping (SRD) method, a notably larger amount of P source is detected. Insights into current understanding of how the rhizosphere responds to changing agricultural or agriculture-based production may be gained from this Research Topic.

In the present Research Topic, attempts have been made to compile the most suitable approaches for rhizosphere manipulation in various environments to improve agricultural yields. The collection of articles in this Research Topic shall pave the way to disseminate the findings of several research experiments conducted in various parts of the world and translate them into the field. The ultimate goal of laboratory and microcosm studies is to translate in the farmer’s field.

## Author contributions

IPS: Investigation, Writing – original draft, Writing – review & editing. KG: Writing – review & editing. NK: Visualization, Writing – review & editing.

